# Development and validation of the Mental Toughness Scale for Chinese Snow Sports Athletes: a mixed-methods study

**DOI:** 10.3389/fpsyg.2026.1905613

**Published:** 2026-09-10

**Authors:** Huanyu Gao, Yangyang Ren, Yimeng Qin, Yuxiang Liang, Dawei Cao, Lianzhong Cao

**Affiliations:** 1 Shenyang Sport University, Shenyang, Liaoning, China; 2 Huaibei Normal University, Huaibei, Anhui, China; 3 Harbin Sport University, Harbin, Heilongjiang, China

**Keywords:** athletes, grounded theory, mental toughness, mixed-methods study, psychometric validation, scale development, snow sports

## Abstract

**Introduction:**

Mental toughness is a key psychological resource that enables athletes to sustain goal-directed engagement, regulate internal states, and maintain consistent performance under pressure, adversity, and uncertainty. Snow sports are strongly influenced by weather and snow conditions, take place in complex training and competition environments, and involve varying degrees of risk. These characteristics require athletes to cope with stress, maintain attentional control, and adapt to changing performance conditions. Existing measures of mental toughness have been developed mainly for general athletic populations and may not fully capture the sport-specific characteristics and psychological demands of snow sports athletes. Accordingly, the present study aimed to develop and validate the Mental Toughness Scale for Chinese Snow Sports Athletes.

**Methods:**

This study employed an exploratory sequential mixed-methods design. In Study 1, semi-structured interviews were conducted with 14 elite Chinese snow sports athletes. Interview data were analyzed using grounded theory coding to identify the core dimensions of mental toughness. In Study 2, an initial item pool was generated from qualitative findings and relevant literature. Items were refined and screened through Delphi consultation, a pilot survey, item analysis, item-total correlation analysis, and exploratory factor analysis, yielding 148 valid pilot responses. In Study 3, confirmatory factor analysis, internal consistency reliability testing, and convergent and discriminant validity assessments were conducted using 342 valid main survey responses.

**Results:**

The qualitative findings supported a three-dimensional structure of mental toughness among snow sports athletes, comprising achievement motivation, positive cognition, and adaptive coping. After item refinement and structural exploration, a 20-item scale representing these three dimensions was finalized. Confirmatory factor analysis supported the three-factor model and indicated good model fit: χ^2^/df = 1.456, RMSEA = 0.037, CFI = 0.982, and TLI = 0.979. The total scale showed strong internal consistency, with a Cronbach’s α coefficient of 0.929, and the subscale coefficients ranged from 0.894 to 0.923. Evidence for convergent and discriminant validity met accepted psychometric standards.

**Discussion:**

The Mental Toughness Scale for Chinese Snow Sports Athletes demonstrated a clear three-dimensional structure and promising preliminary psychometric evidence. The scale may serve as a context-specific instrument for assessing mental toughness among snow sports athletes, although its cross-cultural applicability requires further examination.

## Introduction

Snow sports athletes train and compete under considerable environmental uncertainty. Weather, snow conditions, wind speed, and visibility can vary substantially and affect performance. In these settings, errors in judgment, lapses in attention, or emotional dysregulation may increase the risk of technical errors and injury. Injury surveillance data indicate that World Cup skiers and snowboarders face a high risk of injury, with severe injuries being relatively common in certain disciplines (Flørenes et al., 2012). Therefore, performance in snow sports depends not only on physical and technical abilities but also on athletes’ capacity to remain composed, manage fear, and sustain engagement under pressure and uncertainty.

Mental toughness has been identified as an important psychological resource that helps athletes cope with pressure, challenges, and adversity, and it has been linked to athletic performance (Hsieh et al., 2024). However, the conceptual boundaries and dimensional structure of this construct remain debated. Early research largely adopted a personality-trait perspective, conceptualizing mental toughness as a relatively stable disposition characterized by control, commitment, challenge, and confidence under stress (Clough et al., 2002). Another line of research, based on work with elite athletes, emphasized mental toughness as a psychological advantage that enables athletes to cope with the demands of training, competition, and everyday life, while remaining determined, focused, confident, and in control in critical situations (Jones et al., 2002). Subsequent work further suggests that mental toughness involves not only relatively stable individual differences but also context-dependent and state-like features, and that its structure should be examined in relation to specific performance settings (Gucciardi et al., 2009; Gucciardi et al., 2015).

In scale development research, existing mental toughness instruments have provided an important foundation for quantitative studies. The Psychological Performance Inventory (PPI) was one of the earliest instruments used to assess mental toughness in sport (Loehr, 1986). However, subsequent research found that its seven-factor structure showed inadequate model fit in confirmatory factor analysis, indicating limitations in its psychometric support (Middleton et al., 2004). The Psychological Performance Inventory-Alternative (PPI-A), the Mental Toughness Questionnaire-48 (MTQ48), and the Sports Mental Toughness Questionnaire (SMTQ) were later used to assess mental toughness in athletes and advanced empirical research on this construct (Clough et al., 2002; Golby et al., 2007; Sheard et al., 2009). Among these instruments, the SMTQ was developed specifically for athlete populations and identified three dimensions: confidence, constancy, and control. Although these instruments have made important contributions, their measurement content was developed mainly for general athlete populations or broad sport contexts and may not fully capture the specific psychological demands of snow sports.

Sport-specific applicability therefore requires closer attention in the measurement of athletes’ mental toughness. Previous studies developed the Australian Football Mental Toughness Inventory (AfMTI) and the Cricket Mental Toughness Inventory (CMTI) based on qualitative data, suggesting that the expression of mental toughness may differ across sports. Even within team sports, athletes may face distinct competitive tasks, technical demands, and situational pressures (Gucciardi et al., 2009; Gucciardi and Gordon, 2009). Accordingly, mental toughness measures for athletes should account for sport specificity to improve the fit between measurement content and real competitive contexts.

Against this background, the present study adopted a mixed-methods design to develop and validate the Mental Toughness Scale for Chinese Snow Sports Athletes. Study 1 used semi-structured interviews and grounded theory analysis to identify the core dimensions of mental toughness among snow sports athletes. Study 2 generated items based on the qualitative findings and relevant literature, followed by item refinement through expert consultation and a pilot survey. Study 3 used main survey data to examine the factor structure, internal consistency reliability, convergent validity, and discriminant validity of the scale. This study aims to provide a context-specific measurement instrument for assessing mental toughness, identifying training needs, and supporting subsequent empirical research among snow sports athletes.

## Study 1: identifying the core dimensions of mental toughness among snow sports athletes

The dimensional structure of mental toughness among snow sports athletes has not been sufficiently clarified. Study 1 aimed to identify the core dimensions of mental toughness in this population by drawing on athletes’ lived experiences in training and competition, thereby providing a foundation for subsequent item generation and scale development.

### Methods

#### Formulation of the interview guide

Semi-structured interviews were used to collect qualitative data. The interview guide was developed based on the research objectives, relevant literature on athletes’ mental toughness, and the training and competition contexts of snow sports. The research team responsible for developing the guide consisted of three PhD holders in sport science, two master’s degree holders in sport science, and one associate professor of sport psychology. The team discussed the interview topics, question order, and wording before drafting a preliminary interview guide. Experts in sport psychology and snow sports training were then invited to review the content and wording of the guide. The interview questions and their order were revised according to their feedback.

The final interview guide incorporated questioning techniques from behavioral event interviews and life-history interviews. It focused on athletes’ psychological experiences and coping processes during successful competitions, unsuccessful competitions, high-pressure situations, unexpected training incidents, and critical experiences across their athletic careers. Representative questions included: “Please recall a competition that you considered highly successful or personally meaningful. What psychological factors helped you perform at a high level?”; “Please describe the most challenging or stressful situation you experienced during a major competition. How did you cope with it and remain calm?”; and “Which events in your athletic career tested your determination or beliefs, and how did you overcome them?”

### Participants

Purposive sampling was used to identify the initial participants, and theoretical sampling was subsequently applied during data collection and analysis to supplement the sample. The inclusion criteria were as follows: athletes had won medals in national or higher-level snow sports competitions, had more than 3 years of systematic training experience in snow sports, and were able to provide detailed accounts of pressure experiences and psychological regulation processes during training and competition. Athletes who were unable to complete the interview or could not provide valid interview data due to physical, psychological, or other reasons were excluded from the analysis.

A total of 14 elite Chinese snow sports athletes were included. Participants were recruited from professional training institutions and university sports teams and represented six disciplines: snowboarding, freestyle skiing, alpine skiing, cross-country skiing, ski jumping, and biathlon. Among them, six participants had won medals in international competitions, and eight had won medals in national competitions. All participants provided written informed consent before the interviews, including consent for the publication of the competitive background information reported in Table 1. To avoid direct identification, names and affiliated institutions were not reported.

**Table 1:** Competitive background of interview participants.

Participant ID	Discipline	Representative medal-winning competition
P1	Snowboarding	World Cup Copper Mountain, United States
P2	Snowboarding	World Cup Ruka, Finland
P3	Snowboarding	World Cup Park City, United States
P4	Freestyle skiing	Beijing 2022 Olympic Winter Games, China
P5	Freestyle skiing	World Cup Lake Placid, United States
P6	Freestyle skiing	FIS Freestyle Ski World Championships, Georgia
P7	Alpine skiing	National Alpine Skiing Championships, China
P8	Alpine skiing	14th National Winter Games, China
P9	Cross-country skiing	National Cross-Country Skiing Championships, China
P10	Cross-country skiing	National Cross-Country Skiing Championships, China
P11	Ski jumping	National Ski Jumping Championships, China
P12	Ski jumping	14th National Winter Games, China
P13	Biathlon	14th National Winter Games, China
P14	Biathlon	14th National Winter Games, China

### Interview procedures and data collection

Before the interviews, the research team contacted participants by telephone or online platforms to explain the study purpose, interview topics, confidentiality arrangements, and audio-recording procedures. The interview format, time, and location were then arranged according to each participant’s training schedule, competition schedule, and personal availability. Formal interviews were conducted one-on-one using a semi-structured format. Each interview lasted approximately 30–40 min and was audio-recorded in full after written informed consent had been obtained.

During the interviews, the interviewer followed the interview guide and used appropriate follow-up questions based on participants’ responses. This approach was used to elicit athletes’ lived experiences related to training pressure, competition pressure, major setbacks, injury risk, and critical competitive situations. After each interview, the research team promptly transcribed the audio recordings and checked the transcripts against the interview notes. In total, the interviews with 14 participants yielded approximately 120,000 Chinese characters of textual material, which served as the basis for subsequent grounded theory analysis.

### Grounded theory

Grounded theory is a qualitative research methodology through which concepts and explanatory frameworks are developed from empirical data. It is particularly suitable for research topics with unclear conceptual boundaries and structural dimensions that require further refinement (Glaser and Strauss, 1967). The dimensional structure of mental toughness among snow sports athletes has not yet been fully clarified, and its psychological characteristics need to be identified from athletes’ training and competition experiences. Therefore, this study adopted the procedural grounded theory approach proposed by Strauss and Corbin (1990) to analyze the interview data and identify the core dimensions of mental toughness and their structural relationships.

### Data analysis

After the interview recordings had been transcribed, the research team anonymized all names, institutional affiliations, and personal information unrelated to the study purpose that appeared in the transcripts. The audio recordings, interview notes, and transcripts were then checked for consistency. The cleaned transcripts were imported into NVivo 14.0 for data management and coding.

Data analysis followed the coding procedures of procedural grounded theory, including open coding, axial coding, and selective coding. During open coding, the research team read the interview transcripts line by line, labeled raw statements related to mental toughness, and used constant comparison to generate initial concepts and categories. During axial coding, the team examined conceptual connections among categories and integrated categories with similar meanings or close relationships into higher-order main categories. During selective coding, the team organized the relationships among the main categories around a core category and identified the core dimensions of mental toughness among snow sports athletes.

### Trustworthiness

The trustworthiness of the qualitative findings was enhanced through inter-coder reliability assessment, triangulation, and expert feedback. During the coding process, two doctoral students in sport science participated in the coding in addition to the principal researcher. After the coding principles and operational procedures had been clarified, the three coders independently coded the interview transcripts and cross-checked the core concepts, category labels, and category assignments. The inter-coder reliability assessment indicated a high level of agreement among the three coders (Fleiss’ κ = 0.83). For text segments with coding discrepancies, the research team returned to the original interview materials, reviewed the relevant context, and reached consensus through discussion.

Triangulation and expert feedback were also used to examine and refine the analytical findings. Triangulation was conducted by comparing the interview data with relevant public materials and online media sources, which helped verify and supplement the analytical results. Expert feedback was obtained by presenting the preliminary findings to experts in sport psychology and snow sports training. The experts commented on category labels, conceptual wording, and the interpretation of findings, and the relevant expressions were revised accordingly.

## Results

### Open coding

During open coding, the research team first read the interview transcripts line by line to understand the textual context. Raw statements related to mental toughness were then divided into discrete and analytically meaningful units. The team labeled relevant statements and used constant comparison to generate initial concepts and organize them into open categories. Open coding identified 1,194 coded references and 82 initial nodes, which were ultimately grouped into 22 categories (Table 2).

**Table 2:** Distribution of coded references and categories for mental toughness among snow sports athletes.

No.	Open coding (category)	References, *n*	Axial coding (main category)	Selective coding (core category)
1	Social expectations	44	Achievement motivation	Mental toughness among snow sports athletes
2	Family support	51		
3	Team support	64		
4	Sense of national honor	46		
5	Social encouragement	30		
6	Achievement experience	63		
7	Sport passion	58		
8	Competitive desire	60		
9	Self-confidence	77	Positive cognition	
10	Optimistic tendency	58		
11	Motivational appraisal	30		
12	Emotional awareness	47		
13	Self-evaluation	46		
14	Goal orientation	68		
15	Self-reflection	79		
16	Adaptation to extreme environments	49	Adaptive coping	
17	Coping with competition pressure	73		
18	Emotion regulation	79		
19	Attentional control	56		
20	Courage in facing risks and challenges	31		
21	Help seeking	41		
22	Situational adaptability	44		

### Axial and selective coding

During axial coding, the research team repeatedly compared the relationships among categories and synthesized closely related categories. This process generated three main categories: achievement motivation, positive cognition, and adaptive coping. Achievement motivation refers to the psychological drive through which athletes transform external expectations, social support, national honor, achievement experiences, passion for their sport, and competitive desire into sustained training engagement and athletic pursuit. Positive cognition refers to the psychological process through which athletes make positive appraisals and meaning-based interpretations of their abilities, competitive performance, goal direction, and future outcomes under pressure, after mistakes, and in uncertain situations. Adaptive coping refers to athletes’ capacity to regulate themselves and take effective action in response to environmental changes, competitive pressure, emotional fluctuations, attentional interference, and high-risk situations. During selective coding, the research team further analyzed the relationships among the three main categories and identified “mental toughness among snow sports athletes” as the core category. The correspondence among the 22 categories, three main categories, and the core category is presented in Table 2.

### Theoretical saturation testing

To examine the theoretical saturation of the coding results, the research team analyzed approximately 25% of the interview transcripts that had been reserved in advance, using the same coding procedures. No new concepts, categories, or category relationships emerged from the reserved transcripts, indicating that theoretical saturation had been achieved.

## Study 2: item generation and preliminary scale refinement

### Methods

#### Item generation

Guided by the three main categories of achievement motivation, positive cognition, and adaptive coping, together with the 22 categories derived from Study 1, the research team extracted raw statements from the interview data that reflected the characteristics of mental toughness among snow sports athletes. These statements were then converted into declarative scale items. Item writing followed the principles of clear content, single meaning, concise wording, and contextual relevance to snow sports. After discussion and revision by the research team, an initial item pool of 22 items was generated, including eight items for achievement motivation, seven items for positive cognition, and seven items for adaptive coping.

### Delphi consultation procedure

This study invited 12 experts in sport psychology, sport training, and ice and snow sports to review and refine the initial item pool through Delphi consultation. Experts were selected based on their professional background, research experience, and practical experience. In each round, the experts rated the importance of each dimension and item on a five-point scale and provided suggestions regarding ambiguous wording, overlapping content, unclear dimensional attribution, or insufficient contextual relevance.

The Delphi consultation involved two rounds of feedback. After each round, the research team summarized expert ratings and open-ended comments. Items with a mean importance score of at least 4.00 and a CV of no more than 0.20 were retained after consideration of their conceptual relevance and the experts’ comments. Items were retained, deleted, merged, or reworded accordingly. The revised items were then submitted for the next round of expert consultation until expert opinions reached an acceptable level of agreement. After the Delphi consultation, a pilot version of the Mental Toughness Scale for Chinese Snow Sports Athletes was developed for the pilot survey.

### Pilot survey participants

Participants in the pilot survey were Chinese snow sports athletes who met the inclusion criteria and were recruited through sports teams, training institutions, and sport universities. The represented disciplines included snowboarding, freestyle skiing, cross-country skiing, alpine skiing, and ski jumping. The inclusion criteria were as follows: participants had systematic training experience in snow sports, were able to understand the questionnaire content and complete it independently, and voluntarily participated in the survey with informed consent.

The pilot questionnaire was administered through on-site distribution and an online questionnaire platform. For a small number of participants who were unable to complete and submit the questionnaire independently either on site or online, the research team explained the response procedure by telephone but did not provide guidance on specific item responses. A total of 150 questionnaires were distributed. After questionnaires with incomplete responses, obvious response patterns, or logical inconsistencies had been excluded, 148 valid responses were retained. Questionnaire data were processed anonymously, and all participants completed the survey voluntarily.

Among the respondents, male athletes accounted for 55.4% and female athletes for 44.6%. In terms of competitive level, National Second-Class Athletes, a classification within China’s athlete ranking system, represented the largest proportion, accounting for 41.2%. Training experience was mainly concentrated in the range of 4–6 years, accounting for 45.9%. Among the represented disciplines, alpine skiing athletes accounted for the highest proportion, at 23.0%.

### Statistical analysis

SPSS 27.0 was used to analyze the 148 valid pilot responses. Item screening was performed using item analysis, item-total correlation analysis, internal consistency testing, and exploratory factor analysis. For item analysis, total scores on the pilot questionnaire were ranked from highest to lowest. Respondents in the upper 27% and lower 27% were assigned to the high-score and low-score groups, respectively. Independent-samples *t*-tests were then used to compare item scores between the two groups and evaluate item discrimination. Item-total correlation analysis was conducted to examine the associations between each item score and the total score of the pilot questionnaire. Items with correlation coefficients below 0.50 were considered for deletion. Internal consistency was assessed using Cronbach’s α coefficients, and item quality was further evaluated by examining changes in Cronbach’s α after item deletion.

Before exploratory factor analysis, the Kaiser–Meyer–Olkin test and Bartlett’s test of sphericity were conducted to determine whether the data were suitable for factor analysis. Because the dimensions of mental toughness may be correlated, principal axis factoring was used to extract latent factors, and Promax oblique rotation was applied to identify the factor structure. Items were retained if they met the following criteria: communalities of at least 0.40, primary factor loadings of at least 0.50, cross-loadings below 0.40, and clear theoretical consistency between the item content and its assigned dimension.

## Results

### Delphi consultation results

In both rounds of consultation, 12 questionnaires were distributed and all were returned, yielding a response rate of 100% in each round. The expert authority coefficients were 0.846 in the first round and 0.875 in the second round, indicating a high level of expert authority. The expert coordination analysis showed that Kendall’s W values at the dimension and item levels in the second round were 0.516 and 0.581, respectively, both of which were statistically significant. These results indicated good coordination among expert opinions after item revision.

Based on the first-round results, the item derived from the “social encouragement” category had a mean importance score of 3.33 and a coefficient of variation of 0.295 and therefore did not meet the prespecified retention criteria. After considering the quantitative results and expert comments, the item was deleted. In line with expert feedback and the interview data, an item on “injury recovery” was added to the adaptive coping dimension to reflect athletes’ psychological characteristics related to actively engaging in rehabilitation and returning to training and competition after injury. The second round of expert consultation showed that all revised items met the retention criteria. After two rounds of Delphi consultation, a 22-item pilot version of the Mental Toughness Scale for Chinese Snow Sports Athletes was developed.

### Item analysis and item-Total correlation analysis

The item analysis results showed that all 22 pilot items differed significantly between the high-score and low-score groups, and the critical ratio (CR) values for all items were greater than 3. These findings indicated that the pilot questionnaire items had good discriminatory power. Therefore, no items were deleted at the item analysis stage.

Item-total correlation analysis showed that all items except “situational adaptability” were significantly and positively correlated with the total score of the pilot questionnaire and met the predetermined retention criterion. The correlation coefficient between the “situational adaptability” item and the total score of the pilot questionnaire was 0.463, which was below the retention criterion of 0.50; therefore, this item was deleted. Internal consistency testing showed that, after this item was deleted, the Cronbach’s α coefficients for each dimension and the total scale were all above 0.80, indicating good internal consistency among the retained items. Finally, the remaining 21 items were entered into exploratory factor analysis.

### Exploratory factor analysis and item refinement

Exploratory factor analysis was conducted on the remaining 21 items. The Kaiser–Meyer–Olkin (KMO) measure was 0.926, and Bartlett’s test of sphericity was statistically significant, χ^2^ = 2408.702, df = 210, *p* < 0.001, indicating that the data were suitable for factor analysis. The communalities of all items were greater than 0.40, suggesting that the retained items were adequately explained by the factor structure. Principal axis factoring was used to extract factors, followed by Promax oblique rotation. The eigenvalues of the first three factors were greater than 1, explaining 47.393, 13.249, and 10.052% of the total variance, respectively, with a cumulative variance explained of 70.694%.

The rotated factor loading matrix showed that Factor 1, Factor 2, and Factor 3 corresponded to achievement motivation, positive cognition, and adaptive coping, respectively (Table 3). Except for the item corresponding to “emotional awareness,” all items showed high loadings on their assigned factors and low cross-loadings. The item corresponding to “emotional awareness” had loadings of 0.546 and 0.598 on achievement motivation and positive cognition, respectively, both exceeding 0.40 and indicating a clear cross-loading. To ensure the clarity of the factor structure, this item was deleted.

**Table 3:** Rotated factor loadings and communalities of the Mental Toughness Scale for Chinese Snow Sports Athletes.

No.	Item label	Factor 1 Achievement motivation	Factor 2 Positive cognition	Factor 3 Adaptive coping	Communality
1	Social expectations	0.792			0.661
2	Family support	0.729			0.575
3	Team support	0.718			0.636
4	Sense of national honor	0.808			0.739
5	Achievement experience	0.767			0.680
6	Sport passion	0.833			0.757
7	Competitive desire	0.745			0.626
8	Self-confidence		0.748		0.698
9	Optimistic tendency		0.782		0.730
10	Motivational appraisal		0.796		0.705
11	Emotional awareness	0.546	0.598		0.73
12	Self-evaluation		0.804		0.776
13	Goal orientation		0.810		0.707
14	Self-reflection		0.821		0.729
15	Adaptation to extreme environments			0.770	0.770
16	Coping with competition pressure			0.832	0.729
17	Emotion regulation			0.741	0.707
18	Attentional control			0.851	0.755
19	Courage in facing risks and challenges			0.788	0.661
20	Help seeking			0.763	0.727
21	Injury recovery			0.843	0.747

Only factor loadings with absolute values ≥0.40 are presented.

After item refinement based on item analysis, item-total correlation analysis, and exploratory factor analysis, a final three-dimensional structure consisting of 20 items was formed. Achievement motivation included seven items, positive cognition included six items, and adaptive coping included seven items.

## Study 3: validation of the final scale

### Methods

#### Formal survey participants

Chinese snow sports athletes were recruited through sports teams, training institutions, and sport universities for the formal survey to examine the factor structure and psychometric properties of the 20-item scale developed in Study 2. Participants were required to have systematic training experience in snow sports, be able to understand the questionnaire content and complete it independently, and participate voluntarily in the survey with informed consent. The formal survey sample was independent of the pilot survey sample, and participants from the pilot survey were not included in the formal survey analysis.

A total of 350 formal survey questionnaires were distributed, and 345 were returned. After data screening, questionnaires with incomplete responses, missing key demographic information, or logical inconsistencies were excluded, yielding 342 valid responses (Table 4). In the valid sample, male athletes accounted for 56.7% and female athletes for 43.3%. In terms of competitive level, National Second-Class Athletes represented the largest proportion, accounting for 39.5%. Training experience was mainly concentrated in the range of 4–6 years, accounting for 47.1%. Freestyle skiing and cross-country skiing athletes represented relatively large proportions, accounting for 22.2 and 21.6%, respectively. Questionnaire data were processed anonymously, and all participants completed the survey voluntarily.

**Table 4:** Basic demographic characteristics of respondents in the formal survey.

Variable	Category	*n*	%
Gender	Male	194	56.7
	Female	148	43.3
Competitive level	International Master of Sport	17	5.0
	National Master of Sport	35	10.2
	National first-class athlete	77	22.5
	National second-class athlete	135	39.5
	Unranked athlete	78	22.8
Training experience	3 years or less	124	36.3
	4–6 years	161	47.1
	7 years or more	57	16.7
Discipline	Freestyle skiing	76	22.2
	Snowboarding	60	17.5
	Alpine skiing	60	17.5
	Cross-country skiing	74	21.6
	Ski jumping	28	8.2
	Biathlon	26	7.6
	Nordic combined	18	5.3

Competitive-level categories are based on China’s athlete technical grading system. All participants were registered athletes within China’s competitive sports system and were receiving systematic training at the time of the survey. “Unranked” indicates that an athlete had not obtained an official technical grade at the time of the survey.

### Measures

The 20-item version of the Mental Toughness Scale for Chinese Snow Sports Athletes developed in Study 2 was used in the formal survey. All items were rated on a 5-point Likert scale, with scores ranging from 1 to 5. The full item list is provided in Supplementary Table S1. Higher scores indicated higher levels of mental toughness among athletes.

### Statistical analysis

SPSS 27.0 and AMOS 24.0 were used to analyze the formal survey data. Descriptive statistics were used to summarize participants’ demographic and sport-related characteristics. Confirmatory factor analysis was conducted to examine the fit of the 20-item three-factor structure to the formal survey data. Model fit was evaluated using χ^2^/df, RMSEA, CFI, TLI, and IFI. Convergent validity was assessed using standardized factor loadings, composite reliability, and average variance extracted. Discriminant validity was examined using the Fornell–Larcker criterion by comparing the square root of the average variance extracted for each factor with the correlations among factors. A multiple-indicator multiple-cause (MIMIC) model was used to examine uniform differential item functioning across snow sport disciplines (Woods, 2009). Internal consistency reliability was evaluated using Cronbach’s α and McDonald’s ω coefficients.

## Results

### Confirmatory factor analysis

**Figure 1:**
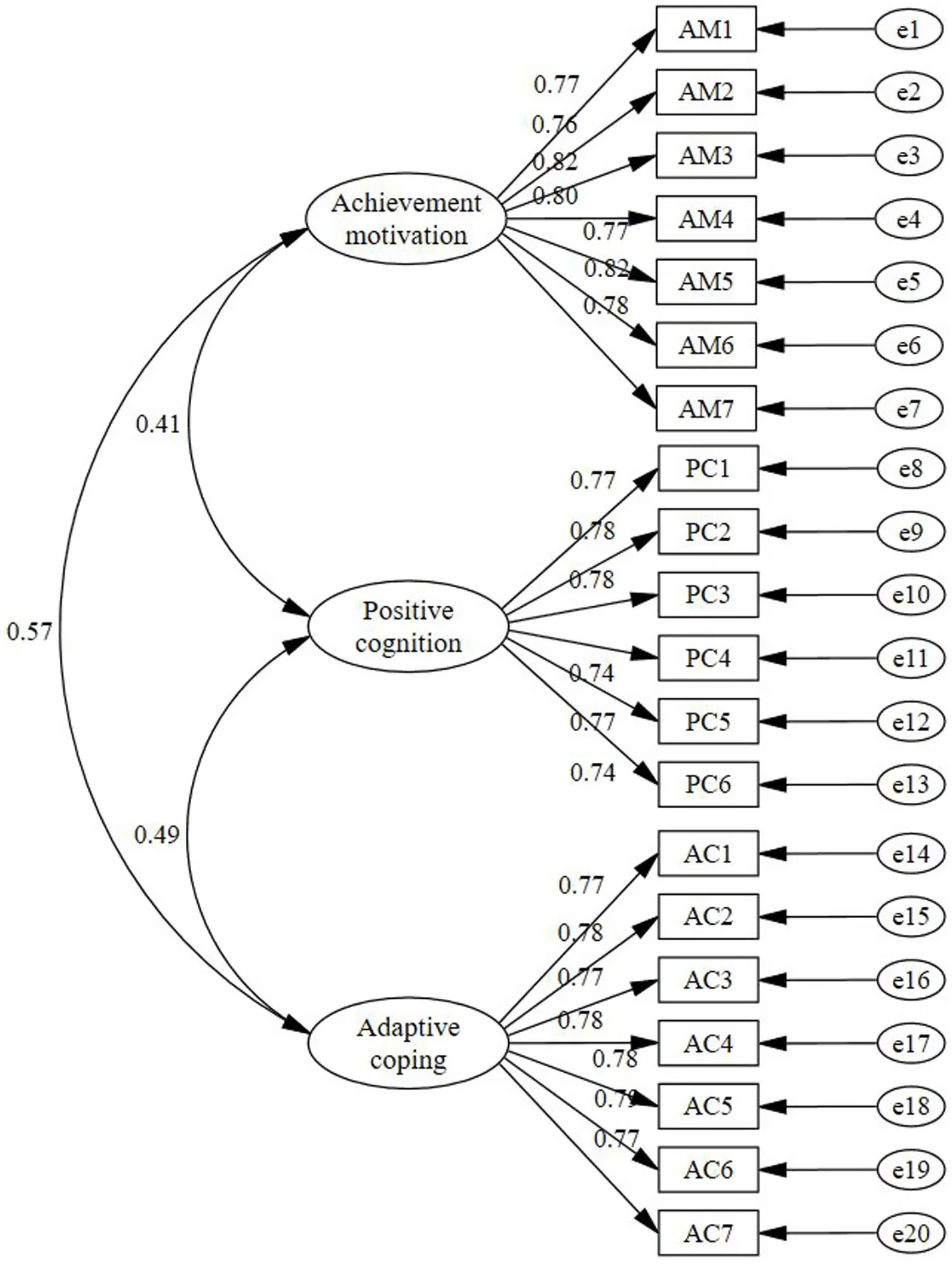
Standardized three-factor confirmatory factor analysis model of the Mental Toughness Scale for Chinese Snow Sports Athletes.

Using the 342 valid formal survey responses, confirmatory factor analysis was conducted to test the 20-item three-factor structure. The model included three first-order factors: achievement motivation, positive cognition, and adaptive coping. Each item was specified to load only on its assigned latent variable, and the three latent variables were allowed to correlate with one another. The results showed that the three-factor model fit the formal survey data well, and all fit indices reached acceptable standards, supporting the three-factor structure of the scale (Table 5 and Figure 1).

**Table 5:** Fit indices of the confirmatory factor analysis model.

Model	χ^2^/df	GFI	AGFI	NFI	IFI	TLI	CFI	RMSEA
Three-factor model	1.456	0.936	0.919	0.944	0.982	0.979	0.982	0.037

### Uniform differential item functioning across snow sport disciplines

The MIMIC model showed a good fit to the data, χ^2^/df = 1.192, CFI = 0.988, TLI = 0.986, IFI = 0.988, and RMSEA = 0.024. After the Benjamini–Hochberg correction, none of the 20 items showed significant uniform differential item functioning across snow sport disciplines (all adjusted *p*-values >0.05). The injury-recovery item likewise showed no uniform DIF, Δχ^2^(6) = 3.89, adjusted *p* = 0.932. Complete item-level results are presented in Supplementary Table S2.

### Convergent validity

Convergent validity was examined using standardized factor loadings, composite reliability (CR), and average variance extracted (AVE). The results showed that the standardized factor loadings of all items were above 0.70, indicating that the items adequately represented their assigned latent variables (Table 6). The CR values for achievement motivation, positive cognition, and adaptive coping were 0.923, 0.894, and 0.914, respectively, all exceeding 0.70. The AVE values were 0.632, 0.585, and 0.604, respectively, all exceeding 0.50. These results supported the convergent validity of the three dimensions of the scale.

**Table 6:** Results of convergent validity testing for the Mental Toughness Scale for Chinese Snow Sports Athletes.

Dimension	Number of items	Standardized factor loading range	Composite reliability (CR)	Average variance extracted (AVE)
Achievement motivation	7	0.764–0.824	0.923	0.632
Positive cognition	6	0.740–0.780	0.894	0.585
Adaptive coping	7	0.766–0.793	0.914	0.604

### Discriminant validity

Discriminant validity was examined by comparing the square root of the AVE for each dimension with the correlations among dimensions. The results showed that the square roots of the AVE values for achievement motivation, positive cognition, and adaptive coping were higher than their correlations with the other dimensions, supporting the relative distinctiveness of the latent variables in the three-dimensional structure (Table 7).

**Table 7:** Results of discriminant validity testing for the Mental Toughness Scale for Chinese Snow Sports Athletes.

Dimension	Achievement motivation	Positive cognition	Adaptive coping
Achievement motivation	0.795		
Positive cognition	0.412**	0.765	
Adaptive coping	0.568**	0.487**	0.777

Diagonal values represent the square roots of AVE values; off-diagonal values represent correlations among latent variables. **p < 0.01.

### Internal consistency reliability

Internal consistency reliability was assessed using Cronbach’s α and McDonald’s ω. The Cronbach’s α and McDonald’s ω coefficients for the total scale and all three dimensions were above 0.80, indicating good internal consistency. Further analysis showed that the ranges of Cronbach’s α if item deleted were 0.908–0.913 for achievement motivation, 0.873–0.879 for positive cognition, and 0.900–0.902 for adaptive coping. These values were not higher than the overall α coefficients of their corresponding dimensions. These results indicated that the 20-item scale had good internal consistency and that each item contributed stably to its assigned dimension (Table 8).

**Table 8:** Internal consistency reliability of the Mental Toughness Scale for Chinese Snow Sports Athletes.

Dimension	Number of items	Cronbach’s α	McDonald’s ω	Cronbach’s α if item deleted
Achievement motivation	7	0.923	0.923	0.908–0.913
Positive cognition	6	0.894	0.892	0.873–0.879
Adaptive coping	7	0.914	0.914	0.900–0.902
Total scale	20	0.929	0.954	—

## Discussion

### The three-factor structure of mental toughness among snow sports athletes

The results supported a three-factor structure of mental toughness among snow sports athletes, comprising achievement motivation, positive cognition, and adaptive coping. This structure suggests that mental toughness among snow sports athletes can be understood from three interrelated aspects: goal engagement, pressure appraisal, and action regulation.

Achievement motivation reflects the motivational foundation that enables athletes to maintain goal engagement and sustained involvement in training. This dimension includes not only personal achievement, sport passion, and competitive desire but also family support, team support, social expectations, and a sense of national honor. This finding suggests that the psychological engagement of snow sports athletes is not derived solely from internal personal characteristics but is also shaped by team responsibility and national competitive tasks.

Positive cognition reflects how athletes appraise pressure, mistakes, and uncertain situations. Maintaining self-confidence, optimism, goal orientation, and self-reflection during complex courses, changing weather conditions, and competition mistakes may help athletes sustain stable judgment and continuous technical execution.

Adaptive coping reflects athletes’ capacity to regulate their internal states and continue performing sport-specific tasks under pressure. Snow sports are characterized by low-temperature environments, complex courses, high-speed movement on snow, fall risk, and rapidly changing competitive situations. Therefore, athletes need not only to withstand pressure but also to regulate emotions rapidly, control attention, cope with risks, and restore their competitive state under pressure.

Compared with existing mental toughness models, the proposed structure retains established elements while reorganizing them around the psychological demands of snow sports. Achievement motivation extends commitment and constancy beyond personal persistence by incorporating socially embedded sources of sustained engagement, including team support, social expectations, and national responsibility. Positive cognition integrates confidence with athletes’ appraisal and reflection under pressure, after mistakes, and in uncertain situations. Adaptive coping broadens the concept of control from internal regulation to effective responses to environmental changes, injury risk, attentional interference, and competition pressure. Thus, the three-dimensional structure does not replace established models but contextualizes and integrates their motivational, cognitive, and regulatory components within snow sports.

### Contextual adaptation of mental toughness measurement

The present scale addresses the tension between general measurement and context-specific adaptation in the assessment of mental toughness. Existing instruments have provided an important foundation for mental toughness research, but they differ in dimensional structure, target populations, and psychometric properties (Farnsworth et al., 2022). The Mental Toughness Index (MTI) provides a brief assessment of overall mental toughness in stressful and challenging situations (Gucciardi et al., 2015). Cross-cultural adaptation research on the MTI among Brazilian athletes suggests that when mental toughness instruments are applied to different cultural contexts and athletic populations, linguistic adaptation, structural stability, and validity evidence need to be re-examined (Moreira et al., 2021). Research on the 10-item Mental Toughness Questionnaire (MTQ10) also indicates that brief general instruments have practical advantages, but cross-cultural applications still require attention to method effects and measurement stability (Dagnall et al., 2019; Denovan et al., 2024).

Taken together, existing instruments are useful for cross-group comparisons and overall assessment of mental toughness, but they may not fully capture the key psychological content embedded in specific sport contexts. The Mental Toughness Scale for Chinese Snow Sports Athletes retains core elements such as goal persistence, self-confidence, positive appraisal, emotion regulation, and coping with pressure, while also incorporating sport-specific content such as adaptation to extreme environments, courage in facing risks and challenges, injury recovery, and coping with competition pressure. The MIMIC analysis showed that none of the 20 items exhibited uniform DIF across the snow sport disciplines represented in the sample. This finding suggests that the items functioned comparably across the sampled snow sport disciplines after differences in the underlying factor levels were taken into account. Therefore, the scale can be understood as a context-specific measure of mental toughness among snow sports athletes.

### Practical implications for dimension-based assessment

The dimension scores provide coaches and sport psychology practitioners with a structured basis for identifying which aspects of an athlete’s mental toughness may require further examination (Gardner, 2019; Jones et al., 2007). Their practical value lies not in assigning athletes a general label, but in generating focused hypotheses that can guide subsequent assessment. A comparatively low score in one dimension can therefore be used to formulate more specific questions about the athlete’s current psychological functioning and the situations in which difficulties are most likely to occur (Sarkar and Fletcher, 2014).

Achievement motivation scores may prompt examination of whether an athlete’s commitment and competitive engagement remain stable across routine training, intensive preparation, competition, and performance setbacks (Clancy et al., 2016; Jones et al., 2007). Positive cognition scores may guide inquiry into how the athlete interprets mistakes and stressful events, evaluates performance, and maintains confidence when expected outcomes are not achieved (Fletcher and Sarkar, 2012; Sarkar and Fletcher, 2014). Adaptive coping scores may direct attention to how effectively the athlete regulates thoughts, emotions, attention, and behavior as situational demands increase (Nicholls and Polman, 2007).

Interpretation also requires attention to the relationships among the three dimensions because motivation, cognition, and coping processes may operate jointly in shaping an athlete’s response to pressure and adversity. A score in one dimension should not be considered in isolation, as similar scores may have different meanings depending on the athlete’s broader response pattern, sporting environment, and current circumstances. For example, reduced training engagement may reflect motivational difficulty, but it may also be associated with repeated performance setbacks, physical or emotional fatigue, athlete burnout, injury status, or changes in competitive expectations (Wiese-Bjornstal et al., 1998; Gustafsson et al., 2011). Dimension-based assessment can therefore help identify the psychological domain in which further inquiry should begin, while interviews and behavioral observations are needed to clarify the personal and situational factors underlying the reported score (Jackson, 1986).

### Injury recovery as a context-specific component of adaptive coping

Injury recovery warrants separate consideration as a context-specific component of adaptive coping. Unlike the items derived directly from the initial qualitative categories, the injury-recovery item was added following expert recommendations during the Delphi process and met all retention criteria in the second round. The item also showed a high loading on adaptive coping in the exploratory factor analysis (0.843), indicating that it was empirically aligned with athletes’ broader capacity to manage demanding situations. Its retention therefore reflects both expert judgment regarding the psychological demands of snow sports and empirical evidence from the scale-development process.

The inclusion of injury recovery is particularly relevant to snow sports because injury may interrupt training progression, competitive preparation, and continued participation. Injury surveillance during the Beijing 2022 Olympic Winter Games identified relatively high injury rates in freestyle skiing and snowboarding, while subsequent research has emphasized the importance of continued and methodologically rigorous injury surveillance across snow sports (Soligard et al., 2024; Spörri et al., 2025). During rehabilitation and return to sport, athletes must regulate emotional responses, maintain engagement with rehabilitation, rebuild confidence, and progressively readjust to training and competition demands. Evidence that psychological resilience and related attributes are associated with psychological adjustment during rehabilitation, recovery outcomes, and return to performance further supports the conceptual placement of injury recovery within adaptive coping (Mastroianni et al., 2025).

However, the relevance of injury recovery should be considered in light of the heterogeneity of the sampled disciplines. Snow sport disciplines differ in movement patterns, environmental demands, injury mechanisms, and levels of risk exposure. Nevertheless, discipline-specific differences in injury exposure do not necessarily preclude injury recovery from serving as a shared indicator of adaptive coping. The MIMIC analysis showed that, after differences in latent adaptive coping levels were taken into account, responses to the item did not vary systematically across disciplines. The present findings therefore support the interpretation of injury recovery as a relevant indicator of adaptive coping across the snow sport disciplines represented in the sample, although further validation using larger discipline-specific samples is required.

## Limitations and future research

This study has several limitations. First, the scale was developed and validated using samples of Chinese snow sports athletes. Its applicability to athletes from other countries, cultural contexts, and training systems therefore remains to be established. Future studies should examine the factor structure, reliability, and measurement invariance of the scale across countries and cultural groups.

Second, reliance on self-report questionnaires may introduce response bias and common method variance and may not fully capture the behavioral manifestations of mental toughness. Because the scale was administered at a single time point, its temporal stability and predictive validity were not evaluated. Future studies should use repeated measurements and multiple data sources, such as coach ratings, behavioral observations, and performance data, to examine test–retest reliability and criterion-related validity.

Third, although the participants represented seven snow sport disciplines, the sample sizes within individual disciplines were insufficient for reliable between-discipline comparisons or measurement invariance testing. Future studies should recruit larger discipline-specific samples to examine whether the factor structure and scale scores remain comparable across snow sport disciplines.

Fourth, National Second-Class and unranked athletes accounted for 62.3% of the formal validation sample. The current findings therefore provide less extensive evidence for athletes holding higher technical grades. Future studies should include larger proportions of National First-Class, National Master-Class, and International Master-Class athletes to examine whether the scale demonstrates comparable psychometric properties across technical-grade groups.

Fifth, the initial item pool included only one provisional item for each qualitative category, which limited the comparison of alternative item formulations. Future studies should generate multiple candidate items for each category and further examine their content coverage and psychometric performance in independent samples.

## Data Availability

The original contributions presented in the study are included in the article/Supplementary material, further inquiries can be directed to the corresponding author.
