# Supplementary material for: Development and validation of the Mental Toughness Scale for Chinese Snow Sports Athletes: a mixed-methods study
Source: Front Psychol. 2026 Sep 10;17:1905613. doi: 10.3389/fpsyg.2026.1905613 (PMC13600811; doi:10.3389/fpsyg.2026.1905613)
Supplement: Supplementary file 1 [file Table_1.docx]

**Supplementary Material**

**Development and Validation of the Mental Toughness Scale for Chinese Snow Sports Athletes: A Mixed-Methods Study**

Supplementary contents.

| **Table** | **Content** |
| --- | --- |
| Supplementary Table S1 | Final scale items and reporting translations |
| Supplementary Table S2 | Uniform Differential Item Functioning Across Snow Sport Disciplines |
| Supplementary Table S3 | Interview guide |
| Supplementary Table S4 | Examples of grounded theory coding |
| Supplementary Table S5 | Initial item pool and item retention decisions |
| Supplementary Table S6 | Delphi consultation results |
| Supplementary Table S7 | Delphi item-level ratings and decisions |
| Supplementary Table S8 | Pilot survey sample characteristics |
| Supplementary Table S9 | Pilot item-total correlations and internal consistency diagnostics |
| Supplementary Table S10 | Exploratory factor analysis results |
| Supplementary Table S11 | Internal consistency diagnostics for the final scale in the main survey |

Supplementary Table S1. Final items of the Mental Toughness Scale for Chinese Snow Sports Athletes.

| **Dimension** | **Reporting code** | **Item label** | **English reporting translation** |
| --- | --- | --- | --- |
| achievement motivation | AM1 | social expectations | I feel that expectations from society motivate me to put greater effort into training and competition. |
|  | AM2 | family support | Support from my family makes me more determined and motivated in training and competition. |
|  | AM3 | team support | Help and encouragement from teammates and coaches motivate me to give my best in competition. |
|  | AM4 | sense of national honor | I have a strong sense of mission and continually pursue excellence in training and competition for national honor. |
|  | AM5 | achievement experience | Pursuing a sense of personal accomplishment in training and competition motivates me to keep striving. |
|  | AM6 | sport passion | I am passionate about snow sports, and each training session and competition makes me feel excited and fulfilled. |
|  | AM7 | competitive desire | I am always eager to compete with other athletes and achieve good results. |
| positive cognition | PC1 | self-confidence | I am confident in my abilities during training and competition. |
|  | PC2 | optimistic tendency | Even in adversity, I believe that the situation will improve. |
|  | PC3 | motivational appraisal | When facing challenges, I can motivate myself to persist and complete the task at hand. |
|  | PC4 | self-evaluation | I regularly review my performance in training and competition and objectively evaluate my strengths and weaknesses. |
|  | PC5 | goal orientation | I remain focused on achieving my goals during training and competition. |
|  | PC6 | self-reflection | I often reflect on my performance to identify areas for improvement. |
| adaptive coping | AC1 | Adaptation to extreme environments | I can effectively cope with extreme weather and environmental changes without giving up easily. |
|  | AC2 | coping with competition pressure | When I experience pressure during competition, I can use effective methods to reduce stress and maintain an optimal performance state. |
|  | AC3 | emotion regulation | After a loss or when facing difficulties, I can quickly regulate my emotions and refocus on competition. |
|  | AC4 | attentional control | I can maintain concentration during training and competition and resist external distractions. |
|  | AC5 | courage to face risk and challenge | When facing high-risk challenges in snow sports competitions, I can overcome fear and face the challenge with courage. |
|  | AC6 | help seeking | When needed, I actively seek help from teammates, coaches, or other people. |
|  | AC7 | injury recovery | After an injury, I can actively develop and implement a rehabilitation plan to recover physically and return to competition. |

Note. All items are positively worded and were administered in Chinese using a 5-point Likert response format.

Supplementary Table S2. Uniform Differential Item Functioning Across Snow Sport Disciplines

| **item** | **Item Wording** | **Dimension** | **Δχ²(6)** | **p** | **BH-Adjusted p** | **DIF** |
| --- | --- | --- | --- | --- | --- | --- |
| AM1 | Social expectations | Achievement motivation | 4.191 | 0.651 | 0.932 | No |
| AM2 | Family support | Achievement motivation | 7.041 | 0.317 | 0.932 | No |
| AM3 | Team support | Achievement motivation | 4.701 | 0.583 | 0.932 | No |
| AM4 | Sense of national honor | Achievement motivation | 7.500 | 0.277 | 0.932 | No |
| AM5 | Achievement experience | Achievement motivation | 1.862 | 0.932 | 0.932 | No |
| AM6 | Sport passion | Achievement motivation | 5.431 | 0.490 | 0.932 | No |
| AM7 | Competitive desire | Achievement motivation | 3.862 | 0.695 | 0.932 | No |
| PC1 | Self-confidence | Positive cognition | 1.981 | 0.921 | 0.932 | No |
| PC2 | Optimistic tendency | Positive cognition | 6.283 | 0.392 | 0.932 | No |
| PC3 | Motivational appraisal | Positive cognition | 8.358 | 0.213 | 0.932 | No |
| PC4 | Self-evaluation | Positive cognition | 4.743 | 0.577 | 0.932 | No |
| PC5 | Goal orientation | Positive cognition | 2.799 | 0.834 | 0.932 | No |
| PC6 | Self-reflection | Positive cognition | 4.113 | 0.661 | 0.932 | No |
| AC1 | Adaptation to extreme environments | Adaptive coping | 3.753 | 0.710 | 0.932 | No |
| AC2 | Coping with competition pressure | Adaptive coping | 4.398 | 0.623 | 0.932 | No |
| AC3 | Emotion regulation | Adaptive coping | 6.405 | 0.379 | 0.932 | No |
| AC4 | Attentional control | Adaptive coping | 6.124 | 0.409 | 0.932 | No |
| AC5 | Courage in facing risks and challenges | Adaptive coping | 2.729 | 0.842 | 0.932 | No |
| AC6 | Help seeking | Adaptive coping | 2.281 | 0.892 | 0.932 | No |
| AC7 | Injury recovery | Adaptive coping | 3.892 | 0.691 | 0.932 | No |

Supplementary Table S3. Semi-structured interview guide.

| **Interview section** | **English reporting translation** |
| --- | --- |
| Opening question | Please describe your athletic background and experience. |
| Behavioral event interview | Please recall a competition that you considered highly successful or personally meaningful. What psychological factors helped you maintain a positive state and perform at a high level? |
|  | Please recall a competition that you experienced as a major failure. How did you feel at that time, and how did you recover emotionally and adjust your mindset? |
|  | Please describe the most challenging or stressful situation you encountered during a major competition. What do you think caused this situation or pressure, and how did you cope with it and remain calm? |
|  | Please describe an unexpected incident you encountered during training. How did you respond and adjust at that time? |
| Life-history interview | Which events in your athletic career tested your determination or beliefs, and how did you ultimately overcome them? |
|  | How do you maintain your passion and motivation for sport? What psychological factors have helped you sustain high-level athletic performance? |
|  | Do you have any regular routines or daily practices that help you adapt to competitive environments and cope with pressure? If so, why are they helpful? |

Note. The guide combined behavioral event interview and life-history interview techniques.

Supplementary Table S4. Examples of grounded theory coding.

| **Data source** | **Raw excerpt** | **Initial labels** | **Open category** | **Main category** |
| --- | --- | --- | --- | --- |
| 访谈资料  interview excerpt | “那天比赛结束后，我真的很激动，因为我知道自己做到了……每一次从空中飞跃，每一次成功地降落，都给我一种成就感。这种感觉，可能是我一直追求的。” | 感到激动；付出得到回报；爱上了滑雪；滑雪获得成就感 | 成就体验 achievement experience | 成就动机 achievement motivation |
|  | “我能感受到，每一次跳跃，下面都有数以万计的观众目不转睛地盯着我……我试着将注意力转移到技术上……深呼吸，尽量放松自己。” | 观众压力；背负期望与祝福；转移注意力；全身心投入；放松自己 | 赛事压力应对 coping with competition pressure; 注意力控制 attentional control | 适应性应对 adaptive coping |
|  | “我会找一个安静的地方，深呼吸，然后默默地告诉自己……我有能力去面对……只专注于自己的比赛。” | 提醒自己；有能力去面对；专注于比赛；激励自己 | 自信 self-confidence; 注意力控制 attentional control | 积极认知 positive cognition; 适应性应对 adaptive coping |
| 公开访谈资料  Public interview excerpt | “到今年9月，就是我滑单板的第20年了……我觉得这是因为对这个项目的喜爱在支持我……它就是我的生活方式了。” | 项目热爱；努力坚持；感到放松；一种生活方式 | 项目热情 sport passion | 成就动机 achievement motivation |
|  | “我觉得滑雪让我学会了不放弃，要坚强，从哪摔倒要从哪站起来，不断地去挑战自我……” | 坚韧不拔的精神；挑战自我；追寻快乐 | 竞争欲望 competitive desire; 风险挑战勇气 courage to face risk and challenge | 成就动机 achievement motivation; 适应性应对 adaptive coping |

Note. Only selected excerpts are presented to illustrate the coding process and reduce participant-identification risk.

Supplementary Table S5. Item pool and retention decisions across the Delphi and pilot phases.

| **Initial code** | **Item label** | **Dimension** | **Decision** |
| --- | --- | --- | --- |
| 1 | social expectations | achievement motivation | Retained |
| 2 | family support |  | Retained |
| 3 | team support |  | Retained |
| 4 | sense of national honor |  | Retained |
| 5 | social encouragement |  | Deleted after Delphi consultation |
| 6 | achievement experience |  | Retained |
| 7 | sport passion |  | Retained |
| 8 | competitive desire |  | Retained |
| 9 | self-confidence | positive cognition | Retained |
| 10 | optimistic tendency |  | Retained |
| 11 | motivational appraisal |  | Retained |
| 12 | emotional awareness |  | Deleted after EFA due to cross-loading |
| 13 | self-evaluation |  | Retained |
| 14 | goal orientation |  | Retained |
| 15 | self-reflection |  | Retained |
| 16 | Adaptation to extreme environments | adaptive coping | Retained |
| 17 | coping with competition pressure |  | Retained |
| 18 | emotion regulation |  | Retained |
| 19 | attentional control |  | Retained |
| 20 | courage to face risk and challenge |  | Retained |
| 21 | help seeking |  | Retained |
| 22 | situational adaptability |  | Deleted after item-total correlation analysis |
| 23 | injury recovery |  | Added after Delphi consultation; retained |

Supplementary Table S6a. Delphi consultation response rate.

| **Round** | **Questionnaires distributed** | **Questionnaires returned** | **Response rate (%)** | **Experts providing suggestions** | **Percentage of experts providing suggestions (%)** |
| --- | --- | --- | --- | --- | --- |
| Round 1 | 12 | 12 | 100 | 8 | 66.7 |
| Round 2 | 12 | 12 | 100 | 3 | 25.0 |

Supplementary Table S6b. Expert authority coefficient.

| **Round** | **Ca** | **Cs** | **Cr** |
| --- | --- | --- | --- |
| 第一轮Round 1 | 0.925 | 0.767 | 0.846 |
| 第二轮Round 2 | 0.933 | 0.817 | 0.875 |

Supplementary Table S6c. Kendall’s coefficient of concordance.

| **Evaluation level** | **Round 1** | | | | **Round 2** | | | |
| --- | --- | --- | --- | --- | --- | --- | --- | --- |
|  | N | W | χ² | P | N | W | χ² | P |
| Scale dimensions | 12 | 0.463 | 11.11 | .004** | 12 | 0.516 | 12.38 | .002** |
| Scale items | 12 | 0.528 | 133.08 | <0.001** | 12 | 0.581 | 146.35 | <0.001** |

Note. N = number of experts; W = Kendall’s coefficient of concordance.

Supplementary Table S7a. First-round Delphi item-level ratings and decisions.

| **No.** | **Indicator** | **Mean** | **Standard deviation** | **（Coefficient of variation）** | **Decision** |
| --- | --- | --- | --- | --- | --- |
| AM | Achievement motivation | 5.00 | 0.000 | 0.000 | Retained |
| PC | Positive cognition | 5.00 | 0.000 | 0.000 | Retained |
| AC | Adaptive coping | 5.00 | 0.000 | 0.000 | Retained |
| AM1 | Social expectations | 4.92 | 0.289 | 0.059 | Retained |
| AM2 | Family support | 4.58 | 0.515 | 0.112 | Retained |
| AM3 | Team support | 5.00 | 0.000 | 0.000 | Retained |
| AM4 | Sense of national honor | 5.00 | 0.000 | 0.000 | Retained |
| AM5 | Social encouragement | 3.33 | 0.985 | 0.295 | Deleted |
| AM6 | Achievement experience | 4.58 | 0.515 | 0.112 | Retained |
| AM7 | Sport passion | 5.00 | 0.000 | 0.000 | Retained |
| AM8 | Competitive desire | 4.92 | 0.289 | 0.059 | Retained |
| PC1 | Self-confidence | 4.83 | 0.577 | 0.119 | Retained |
| PC2 | Optimistic tendency | 4.92 | 0.289 | 0.059 | Retained |
| PC3 | Motivational appraisal | 5.00 | 0.000 | 0.000 | Retained |
| PC4 | Emotional awareness | 4.00 | 0.739 | 0.185 | Retained |
| PC5 | Self-evaluation | 4.92 | 0.289 | 0.059 | Retained |
| PC6 | Goal orientation | 4.92 | 0.289 | 0.059 | Retained |
| PC7 | Self-reflection | 5.00 | 0.000 | 0.000 | Retained |
| AC1 | Adaptation to extreme  environments | 5.00 | 0.000 | 0.000 | Retained |
| AC2 | Coping with competition pressure | 4.83 | 0.389 | 0.081 | Retained |
| AC3 | Emotion regulation | 5.00 | 0.000 | 0.000 | Retained |
| AC4 | Attentional control | 4.92 | 0.289 | 0.059 | Retained |
| AC5 | Courage in facing risks and challenges | 4.83 | 0.389 | 0.081 | Retained |
| AC6 | Help seeking | 4.92 | 0.289 | 0.059 | Retained |
| AC7 | Situational adaptability | 4.75 | 0.452 | 0.095 | Retained |
| AC8 | Injury recovery | - | - | - | Added |

Supplementary Table S7b. Second-round Delphi item-level ratings and decisions.

| **No.** | **Indicator** | **Mean** | **Standard deviation** | **（Coefficient of variation）** | **Decision** |
| --- | --- | --- | --- | --- | --- |
| AM | Achievement motivation | 5.00 | 0.000 | 0.000 | Retained |
| PC | Positive cognition | 5.00 | 0.000 | 0.000 | Retained |
| AC | Adaptive coping | 5.00 | 0.000 | 0.000 | Retained |
| AM1 | Social expectations | 4.92 | 0.289 | 0.059 | Retained |
| AM2 | Family support | 4.92 | 0.289 | 0.059 | Retained |
| AM3 | Team support | 5.00 | 0.000 | 0.000 | Retained |
| AM4 | Sense of national honor | 5.00 | 0.000 | 0.000 | Retained |
| AM6 | Achievement experience | 4.17 | 0.389 | 0.093 | Retained |
| AM7 | Sport passion | 4.92 | 0.289 | 0.059 | Retained |
| AM8 | Competitive desire | 4.83 | 0.577 | 0.119 | Retained |
| PC1 | Self-confidence | 5.00 | 0.000 | 0.000 | Retained |
| PC2 | Optimistic tendency | 4.92 | 0.289 | 0.059 | Retained |
| PC3 | Motivational appraisal | 4.92 | 0.289 | 0.059 | Retained |
| PC4 | Emotional awareness | 4.08 | 0.669 | 0.164 | Retained |
| PC5 | Self-evaluation | 4.83 | 0.577 | 0.119 | Retained |
| PC6 | Goal orientation | 4.92 | 0.289 | 0.059 | Retained |
| PC7 | Self-reflection | 5.00 | 0.000 | 0.000 | Retained |
| AC1 | Adaptation to extreme  environments | 5.00 | 0.000 | 0.000 | Retained |
| AC2 | Coping with competition pressure | 4.92 | 0.289 | 0.059 | Retained |
| AC3 | Emotion regulation | 5.00 | 0.000 | 0.000 | Retained |
| AC4 | Attentional control | 4.92 | 0.289 | 0.059 | Retained |
| AC5 | Courage in facing risks and challenges | 4.83 | 0.577 | 0.119 | Retained |
| AC6 | Help seeking | 4.92 | 0.289 | 0.059 | Retained |
| AC7 | Situational adaptability | 4.92 | 0.289 | 0.059 | Retained |
| AC8 | Injury recovery | 4.17 | 0.389 | 0.093 | Retained |

Supplementary Table S8. Pilot survey sample characteristics.

| **Characteristic** | **Category** | **n** | **Percentage(%)** |
| --- | --- | --- | --- |
| Sex | Male | 82 | 55.4 |
|  | Female | 66 | 44.6 |
| Athlete level | International Master of Sport | 6 | 4.1 |
|  | National Master of Sport | 11 | 7.4 |
|  | National first-class athlete | 38 | 25.7 |
|  | National second-class athlete | 61 | 41.2 |
|  | Unranked athlete | 32 | 21.6 |
| Training years | ≤3 years | 53 | 35.8 |
|  | 4–6 years | 68 | 45.9 |
|  | ≥7 years | 27 | 18.2 |
| Snow sport discipline | Freestyle skiing | 23 | 15.5 |
|  | Snowboarding | 29 | 19.6 |
|  | Alpine skiing | 34 | 23 |
|  | Cross-country skiing | 24 | 16.2 |
|  | Ski jumping | 15 | 10.1 |
|  | Biathlon | 9 | 6.1 |
|  | Nordic combined | 14 | 9.5 |

Supplementary Table S9a. Item-total correlations for achievement motivation in the pilot survey.

| **Item** | **Correlation with the total scale score** | **P** |
| --- | --- | --- |
| Social expectations | 0.603 | <0.001** |
| Family support | 0.589 | <0.001** |
| Team support | 0.689 | <0.001** |
| Sense of national honor | 0.700 | <0.001** |
| Achievement experience | 0.680 | <0.001** |
| Sport passion | 0.668 | <0.001** |
| Competitive desire | 0.618 | <0.001** |

Supplementary Table S9b. Item-total correlations for positive cognition in the pilot survey.

| **Item** | **Correlation with the total scale score** | **P** |
| --- | --- | --- |
| Self-confidence | 0.726 | <0.001** |
| Optimistic tendency | 0.719 | <0.001** |
| Motivational appraisal | 0.665 | <0.001** |
| Emotional awareness | 0.806 | <0.001** |
| Self-evaluation | 0.739 | <0.001** |
| Goal orientation | 0.634 | <0.001** |
| Self-reflection | 0.652 | <0.001** |

Supplementary Table S9c. Item-total correlations for adaptive coping in the pilot survey.

| **Item** | **Correlation with the total scale score** | **P** |
| --- | --- | --- |
| Adaptation to extreme environments | 0.775 | <0.001** |
| Coping with competition pressure | 0.654 | <0.001** |
| Emotion regulation | 0.750 | <0.001** |
| Attentional control | 0.654 | <0.001** |
| Courage in facing risks and challenges | 0.635 | <0.001** |
| Help seeking | 0.760 | <0.001** |
| Situational adaptability | 0.463 | <0.001** |
| Injury recovery | 0.658 | <0.001** |

Supplementary Table S9d. Internal consistency diagnostics for achievement motivation in the pilot survey.

| **Item** | **Scale mean if item deleted** | **Scale variance if item deleted** | **Corrected item–total correlation** | **Cronbach’s α if item deleted** | **Cronbach's Alpha** |
| --- | --- | --- | --- | --- | --- |
| Social expectations | 20.99 | 25.700 | 0.726 | 0.903 | 0.915 |
| Family support | 21.03 | 27.366 | 0.672 | 0.909 | 0.915 |
| Team support | 21.19 | 24.930 | 0.714 | 0.905 | 0.915 |
| Sense of national honor | 21.12 | 24.053 | 0.798 | 0.896 | 0.915 |
| Achievement experience | 21.07 | 25.438 | 0.758 | 0.900 | 0.915 |
| Sport passion | 20.94 | 24.683 | 0.802 | 0.895 | 0.915 |
| Competitive desire | 21.15 | 25.597 | 0.717 | 0.904 | 0.915 |

Supplementary Table S9e. Internal consistency diagnostics for positive cognition in the pilot survey.

| **Item** | **Scale mean if item deleted** | **Scale variance if item deleted** | **Corrected item–total correlation** | **Cronbach’s α if item deleted** | **Cronbach's Alpha** |
| --- | --- | --- | --- | --- | --- |
| Self-confidence | 20.93 | 27.797 | 0.765 | 0.920 | 0.929 |
| Optimistic tendency | 21.16 | 28.327 | 0.799 | 0.916 | 0.929 |
| Motivational appraisal | 21.05 | 29.596 | 0.765 | 0.919 | 0.929 |
| Emotional awareness | 21.11 | 29.232 | 0.738 | 0.922 | 0.929 |
| Self-evaluation | 21.01 | 27.524 | 0.838 | 0.912 | 0.929 |
| Goal orientation | 20.94 | 29.813 | 0.753 | 0.921 | 0.929 |
| Self-reflection | 21.00 | 29.510 | 0.775 | 0.919 | 0.929 |

Supplementary Table S9f. Internal consistency diagnostics for adaptive coping in the pilot survey.

| **Item** | **Scale mean if item deleted** | **Scale variance if item deleted** | **Corrected item–total correlation** | **Cronbach’s α if item deleted** | **Cronbach's Alpha** |
| --- | --- | --- | --- | --- | --- |
| Adaptation to extreme environments | 21.30 | 29.274 | 0.808 | 0.920 | 0.933 |
| Coping with competition pressure | 21.47 | 28.768 | 0.781 | 0.923 | 0.933 |
| Emotion regulation | 21.61 | 29.449 | 0.765 | 0.924 | 0.933 |
| Attentional control | 21.39 | 28.470 | 0.801 | 0.921 | 0.933 |
| Courage in facing risks and challenges | 21.45 | 30.085 | 0.739 | 0.926 | 0.933 |
| Help seeking | 21.43 | 28.273 | 0.794 | 0.921 | 0.933 |
| Injury recovery | 21.60 | 28.527 | 0.798 | 0.921 | 0.933 |

Supplementary Table S9g. Internal consistency coefficients in the pilot survey.

| **dimension** | **Cronbach's Alpha** |
| --- | --- |
| Achievement motivation | 0.915 |
| Positive cognition | 0.929 |
| Adaptive coping | 0.933 |
| Mental toughness | 0.944 |

Supplementary Table S10a. KMO and Bartlett’s test for exploratory factor analysis.

| **Kaiser-Meyer-Olkin** | **Kaiser-Meyer-Olkin** | **0.926** |
| --- | --- | --- |
|  | Approx. Chi-Square | 2408.702 |
| Bartlett | df | 210 |
|  | Sig. | <0.001** |

Supplementary Table S10b. Communalities for the exploratory factor analysis.

| **Item** | **Common factor extraction** | **Item** | **Common factor extraction** | **Item** | **Common factor extraction** |
| --- | --- | --- | --- | --- | --- |
| Social expectations | 0.661 | Optimistic tendency | 0.730 | Emotion regulation | 0.707 |
| Family support | 0.575 | Motivational appraisal | 0.705 | Attentional control | 0.755 |
| Team support | 0.635 | Emotional awareness | 0.730 | Courage in facing risks and challenges | 0.661 |
| Sense of national honor | 0.738 | Self-evaluation | 0.777 | Help seeking | 0.728 |
| Achievement experience | 0.681 | Goal orientation | 0.706 | Situational adaptability | 0.747 |
| Sport passion | 0.757 | Self-reflection | 0.730 |  |  |
| Competitive desire | 0.626 | Adaptation to extreme environments | 0.770 |  |  |
| Self-confidence | 0.698 | Coping with competition pressure | 0.729 |  |  |

Supplementary Table S10c. Eigenvalues and variance explained.

| **Component** | **Initial eigenvalues** | | | **Extraction sums of squared loadings** | | | **Rotation sums of squared loadings** | | |
| --- | --- | --- | --- | --- | --- | --- | --- | --- | --- |
|  | Total | % of variance | Cumulative % | Total | % of variance | Cumulative % | Total | % of variance | Cumulative % |
| 1 | 9.953 | 47.393 | 47.393 | 9.953 | 47.393 | 47.393 | 5.031 | 23.957 | 23.957 |
| 2 | 2.782 | 13.249 | 60.642 | 2.782 | 13.249 | 60.642 | 5.020 | 23.907 | 47.864 |
| 3 | 2.111 | 10.052 | 70.694 | 2.111 | 10.052 | 70.694 | 4.794 | 22.830 | 70.694 |
| 4 | 0.650 | 3.095 | 73.789 |  |  |  |  |  |  |
| 5 | 0.618 | 2.941 | 76.729 |  |  |  |  |  |  |
| 6 | 0.590 | 2.809 | 79.538 |  |  |  |  |  |  |
| 7 | 0.468 | 2.231 | 81.769 |  |  |  |  |  |  |
| 8 | 0.435 | 2.072 | 83.841 |  |  |  |  |  |  |
| 9 | 0.423 | 2.015 | 85.856 |  |  |  |  |  |  |
| 10 | 0.401 | 1.909 | 87.765 |  |  |  |  |  |  |
| 11 | 0.344 | 1.637 | 89.402 |  |  |  |  |  |  |
| 12 | 0.326 | 1.554 | 90.956 |  |  |  |  |  |  |
| 13 | 0.317 | 1.509 | 92.465 |  |  |  |  |  |  |
| 14 | 0.272 | 1.294 | 93.759 |  |  |  |  |  |  |
| 15 | 0.241 | 1.150 | 94.909 |  |  |  |  |  |  |
| 16 | 0.231 | 1.100 | 96.009 |  |  |  |  |  |  |
| 17 | 0.211 | 1.007 | 97.016 |  |  |  |  |  |  |
| 18 | 0.199 | 0.948 | 97.964 |  |  |  |  |  |  |
| 19 | 0.172 | 0.818 | 98.782 |  |  |  |  |  |  |
| 20 | 0.134 | 0.640 | 99.422 |  |  |  |  |  |  |
| 21 | 0.121 | 0.578 | 100 |  |  |  |  |  |  |

Supplementary Table S11a. Internal consistency diagnostics for achievement motivation in the main survey.

| **Item** | **Scale mean if item deleted** | **Scale variance if item deleted** | **Corrected item–total correlation** | **Cronbach’s α if item deleted** | **Cronbach’s α** |
| --- | --- | --- | --- | --- | --- |
| Social expectations | 20.52 | 28.274 | 0.766 | 0.91 | 0.923 |
| Family support | 20.3 | 29.254 | 0.735 | 0.913 | 0.923 |
| Team support | 20.46 | 28.103 | 0.792 | 0.908 | 0.923 |
| Sense of national honor | 20.62 | 27.943 | 0.758 | 0.911 | 0.923 |
| Achievement experience | 20.34 | 28.226 | 0.739 | 0.913 | 0.923 |
| Sport passion | 20.59 | 27.891 | 0.779 | 0.909 | 0.923 |
| Competitive desire | 20.54 | 28.349 | 0.74 | 0.913 | 0.923 |

Supplementary Table S11b. Internal consistency diagnostics for positive cognition in the main survey.

| **Item** | **Scale mean if item deleted** | **Scale variance if item deleted** | **Corrected item–total correlation** | **Cronbach’s α if item deleted** | **Cronbach’s α** |
| --- | --- | --- | --- | --- | --- |
| Self-confidence | 17.86 | 16.156 | 0.721 | 0.874 | 0.894 |
| Optimistic tendency | 17.93 | 15.652 | 0.73 | 0.873 | 0.894 |
| motivational appraisal | 17.83 | 15.639 | 0.729 | 0.873 | 0.894 |
| self-evaluation | 17.94 | 16.304 | 0.689 | 0.879 | 0.894 |
| Goal orientation | 17.94 | 15.92 | 0.724 | 0.874 | 0.894 |
| Self-reflection | 17.88 | 16.505 | 0.699 | 0.878 | 0.894 |

Supplementary Table S11c. Internal consistency diagnostics for adaptive coping in the main survey.

| **Item** | **Scale mean if item deleted** | **Scale variance if item deleted** | **Corrected item–total correlation** | **Cronbach’s α if item deleted** | **Cronbach’s α** |
| --- | --- | --- | --- | --- | --- |
| Adaptation to extreme environments | 21.04 | 25.339 | 0.733 | 0.901 | 0.914 |
| Coping with competition pressure | 20.98 | 25.067 | 0.748 | 0.9 | 0.914 |
| Emotion regulation | 21.03 | 25.14 | 0.728 | 0.902 | 0.914 |
| Attentional control | 21.28 | 24.471 | 0.735 | 0.901 | 0.914 |
| Courage in facing risks and challenges | 21.06 | 25.111 | 0.743 | 0.9 | 0.914 |
| Help seeking | 21.26 | 24.183 | 0.75 | 0.9 | 0.914 |
| Injury recovery | 21.08 | 24.548 | 0.727 | 0.902 | 0.914 |

Supplementary Table S11d. Internal consistency reliability in the formal validation sample.

| **dimension** | **Item** | **Cronbach’s α** | **Range of Cronbach‘s α if item deleted** |
| --- | --- | --- | --- |
| Achievement motivation | 7 | 0.923 | 0.908–0.913 |
| Positive cognition | 6 | 0.894 | 0.873–0.879 |
| Adaptive coping | 7 | 0.914 | 0.900–0.902 |
| Mental toughness | 20 | 0.929 | — |
